# Clonal hematopoiesis in adult pure red cell aplasia

**DOI:** 10.1038/s41598-021-81890-5

**Published:** 2021-01-26

**Authors:** Naohito Fujishima, Junki Kohmaru, Souichi Koyota, Keiji Kuba, Tomoo Saga, Ayumi Omokawa, Yuki Moritoki, Shigeharu Ueki, Fumihiro Ishida, Shinji Nakao, Akira Matsuda, Akiko Ohta, Kaoru Tohyama, Hiroshi Yamasaki, Kensuke Usuki, Yasuhiro Nakashima, Shinya Sato, Yasushi Miyazaki, Yasuhito Nannya, Seishi Ogawa, Kenichi Sawada, Kinuko Mitani, Makoto Hirokawa

**Affiliations:** 1 Division of Blood Transfusion, 44-2 Hiroomote Aza Hasunuma, Akita, 010-8543 Japan; 2 Akita University Bioscience Education and Research Center, Akita, Japan; 3 Department of Biochemistry and Metabolic Science, Akita University Graduate School of Medicine, Akita, Japan; 4 Department of General Internal Medicine and Clinical Laboratory Medicine, Akita University Graduate School of Medicine, Akita, Japan; 5 Department of Biomedical Laboratory Sciences, Shinshu University School of Medicine, Matsumoto, Japan; 6 Department of Cellular Transplantation Biology, Kanazawa University Graduate School of Medicine, Kanazawa, Japan; 7 Department of Hemato-Oncology, International Medical Center, Saitama Medical University, Saitama, Japan; 8 Division of Public Health, Department of Social Medicine, Saitama Medical University, Saitama, Japan; 9 Department of Laboratory Medicine, Kawasaki Medical School, Okayama, Japan; 10 Department of Hematology and Oncology, Kumamoto City Hospital, Kumamoto, Japan; 11 Department of Hematology, NTT Medical Center Tokyo, Tokyo, Japan; 12 Department of Medicine and Bioregulatory Science, Graduate School of Medical Sciences, Kyushu University, Fukuoka, Japan; 13 Department of Hematology, Atomic Bomb Disease and Hibakusha Medicine Unit, Atomic Bomb Disease Institute, Nagasaki University, Nagasaki, Japan; 14 Department of Pathology and Tumor Biology, Graduate School of Medicine, Kyoto University, Kyoto, Japan; 15 Department of Hematology, Nephrology and Rheumatology, Akita University Graduate School of Medicine, Akita, Japan; 16 Department of Hematology and Oncology, Dokkyo Medical University, Tochigi, Japan

**Keywords:** Clinical genetics, Haematological diseases

## Abstract

Idiopathic pure red cell aplasia (PRCA) and secondary PRCA associated with thymoma and large granular lymphocyte leukemia are generally considered to be immune-mediated. The PRCA2004/2006 study showed that poor responses to immunosuppression and anemia relapse were associated with death. PRCA may represent the prodrome to MDS. Thus, clonal hematopoiesis may be responsible for treatment failure. We investigated gene mutations in myeloid neoplasm-associated genes in acquired PRCA. We identified 21 mutations affecting amino acid sequences in 11 of the 38 adult PRCA patients (28.9%) using stringent filtering of the error-prone sequences and SNPs. Four PRCA patients showed 7 driver mutations in *TET2, DNMT3A* and *KDM6A*, and 2 PRCA patients carried multiple mutations in *TET2*. Five PRCA patients had mutations with high VAFs exceeding 0.3. These results suggest that clonal hematopoiesis by stem/progenitor cells might be related to the pathophysiology of chronic PRCA in certain adult patients.

## Introduction

Idiopathic pure red cell aplasia (PRCA) and secondary PRCA not responding to treatments for the underlying diseases in adults are generally considered to be immune-mediated and are treated by immunosuppressive therapy^1,2^. We previously conducted the PRCA2004/2006 study and reported that poor responses to induction therapy and anemia relapse were associated with death^3–6^. Principal causes of death were infections and organ failure. Different outcomes in adult PRCA patients depending on their responses to immunosuppression suggest the heterogeneity of chronic PRCA in adults. Based on previous findings, idiopathic PRCA may be the prodrome to myelodysplastic syndromes^7,8^. In some cases, erythroid hypoplasia/aplasia has been observed in patients with myelodysplastic syndrome (MDS)^9–11^.

Theoretically, there are two potential mechanisms of unresponsiveness to immunosuppression: clonal changes in autoaggressive lymphocytes reacting against erythroid progenitors and clonal hematopoiesis by stem/progenitor cells that have undergone somatic mutations during the disease progression of PRCA. Regarding the former, mutations in the signal transducer and activator of the transcription 3 gene (*STAT3*) were detected in 40% of patients with large granular lymphocyte (LGL) leukemia^12^ and have been found in PRCA^13^, aplastic anemia, and MDS patients^14^. Kawakami et al. recently reported that *STAT3* mutations were detected in 43% of PRCA patients, including LGL leukemia-associated PRCA, idiopathic PRCA and thymoma-associated PRCA^15^. They also reported that *STAT3* mutation-positive patients were less responsive to cyclosporine than those with wild-type *STAT3*. These results appear to support the hypothesis that clonal changes in autoaggressive lymphocytes may be related to the refractoriness of PRCA to immunosuppression.

To test the working hypothesis that clonal hematopoiesis by stem/progenitor cells might be related to the poor responses and outcomes of PRCA patients, we investigated how often clonal hematopoiesis is detected in adult chronic PRCA. We examined gene mutations in myeloid neoplasm-associated genes and found that rare variants or mutations in myeloid neoplasm-associated genes were detected in adult chronic PRCA.

## Results

### Landscape of somatic mutations in PRCA, aplastic anemia, myeloid neoplasm

**Figure 1:**
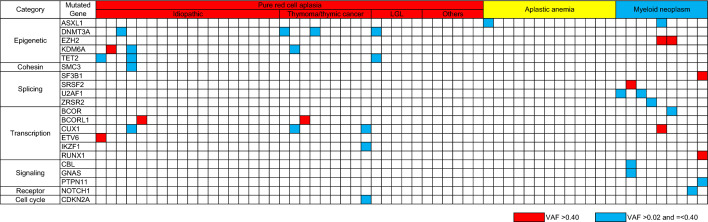
Landscape of gene mutation in PRCA. Overall genomic alterations are shown here. Each row indicates a distinct genomic alteration, and each column shows the results of the respective individual. Upper and lower panels show mutations in coding and noncoding regions, respectively. Mutations with high VAFs (> 0.40) and low VAFs (≤ 0.40) are depicted in red and blue, respectively. None of the 13 healthy control donors showed any genomic alterations.

After stringent filtering of the error-prone sequences and excluding SNPs, the remaining variant sequences were curated as described in the Methods section. We identified at least one genomic mutation in 11 out of 38 patients with PRCA (28.9%), and we observed one mutation in only 1 out of 13 patients with aplastic anemia (*p* = 0.151, Fisher test) and at least one mutation in 7 out of 9 patients with myeloid neoplasms (*p* = 0.0178, Fisher test) (Fig. 1). No gene mutations were detected in 13 healthy control donors (data not shown). The prevalence of gene mutations in idiopathic, thymoma/thymic cancer-associated, LGL-associated, and other causes of PRCA was 28% (5 of 18), 56% (5 of 9), 20% (1 of 5), and 0% (0 of 6), respectively.

### Interpretation of gene mutations in PRCA

We classified the curated mutations into three categories: driver, potential driver, and nondriver. The driver mutations were judged based on the previously reported gene mutations implicated in myeloid malignancies in public databases. With this strategy, we identified 21 mutations affecting amino acid sequences in 11 patients with PRCA (Table 1). Four patients with PRCA had 7 driver mutations, including *TET2*, *DNMT3A*, and *KDM6A*. The affected genes were *TET2, DNMT3A, and KDM6A* with VAFs of 0.03915 to 0.32235. Nine potential driver mutations were found in 8 patients, and the mutated genes were *DNMT3A, SMC3, CUX1, ETV6, BCOR,* and *IKZF* with VAFs of 0.0415 to 0.47025. The five mutations that had been considered irrelevant to myeloid malignancy were found in 4 patients with VAFs of 0.04125–0.501. Collectively, 5 PRCA patients had mutations with very high VAFs exceeding 0.3. Five of 11 patients carried multiple mutations (2–3 genes), and 2 of these 5 patients carried 2 mutations in the same *TET2 gene*. *TET2*, *DNMT3A*, *KDM6A*, *CUX1*, and *BCORL1* mutations were detected frequently in study participants.

**Table 1:** Driver and nondriver mutations in pure red cell aplasia.

Interpretation	Patient/Age	Classification	VAF	Gene	Functional consequence	Amino acid change	Sample
Driver mutations	PR-17–12/73 y	Idiopathic	0.03915	TET2	Frameshift	p.G641fs	PBMCs
			0.1119	TET2	SNV	p.H1904R	
	PR-17–55/81 y	Idiopathic	0.0458	TET2	Frameshift	p.T759fs	PBMCs
	PR-17–50/50 y	Thymoma	0.0486	KDM6A	SNV	p.M1I	PBMCs
	PR-18–76/82 y	LGL-L	0.06955	DNMT3A	Splicing	c.1429+1G>A	WB
			0.32235	TET2	Frameshift	p.P288fs	
			0.0617	TET2	Stopgain	p.R1465X	
Potential driver mutations	PR-17–12/73 y	Idiopathic	0.4336	ETV6	SNV	p.H308N	PBMCs
	PR-17–47/45 y	Idiopathic	0.07685	DNMT3A	SNV	p.T808I	PBMCs
	PR-17–9/60 y	Idiopathic	0.47025	BCORL1	SNV	p.G1391R	PBMCs
	PR-17–55/81 y	Idiopathic	0.10555	SMC3	SNV	p.K684I	PBMCs
			0.0415	CUX1	SNV	p.S1028P	
	PR-17–13/63 y	Thymoma	0.04455	DNMT3A	SNV	p.P799S	PBMCs
	PR-17–50/50 y	Thymoma	0.0903	CUX1	SNV	(c.A1585T)	PBMCs
	PR-18–81/72 y	Thymoma	0.05355	DNMT3A	SNV	p.L373V	WB
	PR-17–51/78 y	Thymic cancer	0.0567	IKZF1	SNV	p.D488N	PBMCs
Nondriver mutations	PR-17–15/56 y	Idiopathic	0.501	KDM6A	SNV	p.A694T	PBMCs
			0.36885	KDM6A	SNV	p.Y80C	
	PR-17–53/48 y	Thymoma	0.40885	BCORL1	SNV	p.E1094G	PBMCs
	PR-17–51/78 y	Thymic cancer	0.04125	CDKN2A	SNV	(c.A217T)	PBMCs
			0.09305	CUX1	SNV	p.Q329R	

LGL-L, large granular lymphocyte leukemia; PBMCs, peripheral blood mononuclear cells; WB, whole blood.

We observed only one mutation of the *ASXL1* gene in a patient with aplastic anemia (Table 2). We also detected frequent gene mutations in 8 of 9 patients with myeloid neoplasms, including multiple driver mutations.

**Table 2:** Driver and nondriver mutations in aplastic anemia and myeloid neoplasms.

Interpretation	Patient/Age	Disease	VAF	Gene	Functional consequence	Amino acid change	Sample
Driver mutations	PR-17–25/72 y	Aplastic anemia	0.1105	ASXL1	Frameshift	p.P808fs	PBMCs
	PR-17–2/82 y	MDS-MLD	0.07735	U2AF1	SNV	p.S34F	WB
	PR-17–39/NA	MDS-MLD	0.49555	SRSF2	SNV	p.P95L	WB
			0.0864	CBL	SNV	p.L380P	
			0.1591	GNAS	SNV	p.R201H	
	PR-17–40/NA	MDS-MLD	0.3232	U2AF1	SNV	p.Q157R	WB
	PR-17–41/NA	AML-MRC	0.04445	ZRSR2	Stopgain	p.K106X	WB
	PR-18–77/70 y	MPN/MDS	0.0324	ASXL1	Frameshift	p.G642fs	WB
	PR-18–78/73 y	MDS-SLD	0.7453	EZH2	SNV	p.D659G	WB
	PR19-95/80 y	MDS-EB2	0.4073	SF3B1	SNV	p.K700E	WB
			0.40385	RUNX1	Stopgain	p.R201X	
			0.3342	PTPN11	SNV	p.N58Y	
	MDS-L2007	Cell line	0.67895	CEBPA	Stopgain	p.Q311X	Cell line
			0.6548	NRAS	SNV	p.G12A	
			0.9569	TP53	Splicing	c.672+1G>A	
Potential driver mutations	PR-18–77/70 y	MPN/MDS	0.74965	CUX1	Frameshift	p.R254fs	WB
	PR-18–78/73 y	MDS-SLD	0.0345	BCOR	Frameshift	p.E1182fs	WB
Nondriver mutations	PR-18–70/NA	MDS-EB1	0.04745	NOTCH1	SNV	p.L1531M	WB

*MDS* myelodysplastic syndrome, *MDS-MLD* MDS with multilineage dysplasia, *AML-MRC* acute myeloid leukemia with myelodysplasia-related changes, *MPN*, myeloproliferative neoplasm, *MDS-SLD* MDS with single lineage dysplasia, *MDS-EB* MDS with excess blasts, *NA* not available, *PBMCs* peripheral blood mononuclear cells, *WB* whole blood.

## Discussion

The present results demonstrate that adult chronic PRCA patients frequently have genomic alterations in myeloid neoplasm-associated genes, which are derived from heterogeneous origins, including common SNPs, rare variant SNPs, and mutations. With regard to the mutations in PRCA, we made two important observations in this study. First, we found 21 distinct mutations of myeloid neoplasm-associated genes in 11 out of 38 adult patients (28.9%) with chronic PRCA, and the 4 patients had driver mutations in *TET2*, *DNMT3A* or *KDM6A*. Second, despite its unknown relevance to disordered hematopoiesis, we found ambiguous and unlikely driver mutations in 4 patients. Interestingly, 5 patients had mutations with high VAFs exceeding 0.3. These two findings suggest the working hypothesis that clonal hematopoiesis by stem/progenitor cells might be related to the pathophysiology of chronic PRCA, at least in a certain proportion of adult patients.

In the present study, we focused on genes implicated in myeloid malignancies and found that mutations in myeloid neoplasm-associated genes in PRCA patients were widely distributed in epigenetics, cohesin, transcription, and cell cycle-related genes. Although *STAT3* mutations are frequently detected in PRCA patients^13,15^, we did not identify *STAT3* mutations in the present study because this gene was not included in the target sequencing panel.

The mutational patterns of PRCA resembled those of myeloid neoplasms. The median age of PRCA patients in this study was 65 years. Jaiswal S, et al. previously reported that age-related clonal hematopoiesis (CHIP) in individuals in their 60 s has been reported in 5.6% (95% confidence interval, 5.0 to 6.3) of the general population, and the majority of these variants occur in epigenetic-related genes, such as *DNMT3A*, *TET2*, and *ASXL1*^16^. This has been confirmed by Genovese et al.^17^. Therefore, it is possible to assume that the mutations of *TET2* and *DNMT3A* in our PRCA patient cohort might reflect the age-related clonal hematopoiesis. The prevalence of genomic alterations in the present PRCA cohort appears to exceed the rate of age-related clonal hematopoiesis, although this remains to be clarified by examining the age-matched healthy controls, since the median age of the healthy control donors in this study was 25.

The limitations of our study were mostly derived from its design. First, we did not have germline controls for analyses. Thus, we could not correctly determine the origins of mutations. Second, a large portion of the samples were analyzed in a retrospective manner, the sample size was too small, and clinical information was limited. Thus, we could not explore the association of the mutations with clinical responses to treatment. Third, we only examined the targeted mutations of 54 myeloid neoplasm-associated genes but not of other genes involved in hematopoiesis. Finally, we could not determine the identified mutations in myeloid lineage cells or lymphoid cells or both in PRCA patients because of the analyzed samples.

The reason why immunosuppression is effective in erythropoietic disorders with genetic alterations in myeloid neoplasm-associated genes is still being debated. Although genomic alterations may become driver mutations that directly affect hematopoiesis, mutational events may also produce mutant proteins that are recognized as altered self^18^. Recently, chronic inflammatory responses have been proposed to reflect the pathophysiology of MDS^19–23^. Moreover, Balasubramanian et al. reported that the genetic alterations found in MDS were detected in 5 typical idiopathic PRCA patients in their cohort^24^, suggesting that our results are consistent with their findings. Another possibility is that immunosuppressants induce the erythroid differentiation of hematopoietic stem/progenitor cells in vitro, as described previously by Sawafuji et al.^25^.

It is generally believed that idiopathic PRCA and secondary PRCA not responding to treatments for the underlying diseases in adults are generally considered to be immune-mediated and are treated with immunosuppressive therapy^1,2^. Our present study has demonstrated that adult chronic PRCA is a heterogeneous hematopoietic disorder with various genetic backgrounds. The pathogenicity or functional significance of the somatic mutations found in the present PRCA patient cohort remains to be elucidated by future experiments.

Molecular genetic testing with next-generation sequencing is a candidate tool for the classification and prognostic evaluation of bone marrow failure, such as MDS and aplastic anemia^26–32^. A prospective study is needed to confirm the present results and clarify the diagnostic and predictive values of genetic variations in myeloid neoplasm-associated genes in acquired PRCA. This project is ongoing in collaboration with the prospective cohort study PRCA2016 currently being conducted in Japan.

## Methods

### Patients

The present study included 38 patients with chronic acquired PRCA (18 idiopathic, 9 thymoma-, 5 LGL leukemia-, 2 systemic lupus erythematosus-, 2 viral infection-, one autoimmune hepatitis-, and one end-stage renal disease-associated PRCA); patients had a median age of 65 years (range: 34–82 years) and were recruited from multiple collaborative centers. The disease status varied among patients. Thirteen patients with idiopathic aplastic anemia had a median age of 68 years (range: 35–88 years), 9 with myeloid neoplasms had a median age of 75 years (range: 70–82 years), and 13 healthy control donors had a median age of 25 years (range: 23–34 years). The diagnostic criteria and classification of PRCA as well as the response criteria for PRCA were based on previous findings^1,3–5^. Aplastic anemia was diagnosed and classified according to previous findings^33,34^. MDS was diagnosed and classified according to the WHO 2017 classification of myeloid neoplasms^35^. This study was approved by the institutional review board of Akita University Graduate School of Medicine and was performed in accordance with the Declaration of Helsinki. Written informed consent was obtained from patients and healthy controls. This study was registered as an observational study at the UMIN Clinical Trials Registry (UMIN-CTR) (UMIN000033866).

### Cell line

The established cell line MDS-L2007 was used to assess the sensitivity of targeted sequencing for myeloid malignancy-associated genes. This cell line was established from a patient with MDS by Matsuoka et al.^36^ and harbored mutations including *NRAS* G12A, *CEBPA* Q311X, and *TET2* F1901fs.

### Mutation analysis

Extracted genomic DNA samples from peripheral blood were subjected to targeted sequencing for 54 myeloid malignancy-associated genes using a MiSeq system and a TruSight Myeloid Sequencing Panel kit according to the manufacturer’s instructions (Illumina). To address the clonal hematopoiesis by stem/progenitor cells that might be related to the pathogenesis and pathophysiology of PRCA, the NGS panel primarily included myeloid malignancy-associated genes. STAT3 gene mutations were not included. In some cases, DNA from peripheral blood mononuclear cells (PBMCs) obtained by density gradient centrifugation was used in the analyses. Germline controls were not included in the present study.

The sensitivity of the mutation analysis was assessed using serial dilutions of the MDS-L2007 cells in healthy control donor cells (Supplementary Fig. 1). To avoid false-positive results, mutations were identified only by the presence of identical gene alterations in two different sequencing runs for each sample because certain gene mutations were detected in one sequencing run but not in the other, particularly when VAFs were not high. Other researchers have established a cutoff value of higher than 0.02 for positive VAFs in age-related clonal hematopoiesis^16^. Thus, we employed a cutoff value for positive variant allele frequencies (VAFs) of higher than 0.03 for assessing the global genetic alterations in patients at the first step of mutation analysis.

Synonymous single nucleotide variants (SNVs) and ambiguous SNVs that were also found in control specimens were excluded. SNVs that were registered in public databases—including the dbSNP (the National Center for Biotechnology Information), jMorp (Japanese Multi Omics Reference Panel), 1000 Genomes Project (IGSR: The International Genome Sample Resource), and ExAC (Exome Aggregation Consortium)—were considered putative SNPs. To obtain the mutations affecting amino acid sequences (potentially relevant to the pathophysiology in chronic PRCA), exonic variants were selected and curated with public databases, including CLINVAR, COSMIC v90, and InterVar^26,27,37^ (Supplementary Data).

### Statistical analysis

Comparisons between different groups were carried out using Fisher’s exact test as appropriate. *P* < 0.05 was considered to indicate statistical significance. Fisher’s exact test p-value calculations were double-checked for accuracy by two independent coresearchers. All statistical analyses were performed using the EZR software program^38^.

## Supplementary Information

Supplementary Figure 1.

Supplementary Data.
