# Supplementary material for: Clonal hematopoiesis in adult pure red cell aplasia
Source: Sci Rep. 2021 Jan 26;11:2253. doi: 10.1038/s41598-021-81890-5 (PMC7838416; doi:10.1038/s41598-021-81890-5)
Supplement: Supplementary file 1 — Supplementary Figure 1. [file 41598_2021_81890_MOESM1_ESM.pdf]

Version 1
2020-09-24

Clonal hematopoiesis in adult pure red cell aplasia

Running title: Clonal hematopoiesis in PRCA

Naohito Fujishima,[1] Junki Kohmaru,[2] Souichi Koyota,[2] Keiji Kuba,[2,3] Tomoo Saga,[4] Ayumi Omokawa,[4] Yuki Moritoki,[4] Shigeharu Ueki,[4] Fumihiro Ishida,[5] Shinji Nakao,[6] Akira Matsuda,[7] Akiko Ohta,[8] Kaoru Tohyama,[9] Hiroshi Yamazaki,[10] Kensuke Usuki,[11] Yasuhiro Nakashima,[12] Shinya Sato,[13] Yasushi Miyazaki,[13] Yasuhito Nannya, [14] Seishi Ogawa, [14] Kenichi Sawada,[15] Kinuko Mitani[16] and Makoto Hirokawa[4]

1. *Division of Blood Transfusion, Akita University Hospital, Akita, Japan*
2. *Akita University Bioscience Education and Research Center, Akita, Japan*
3. *Department of Biochemistry and Metabolic Science, Akita University Graduate School of Medicine, Akita, Japan*
4. *Department of General Internal Medicine and Clinical Laboratory Medicine, Akita University Graduate School of Medicine, Akita, Japan*
5. *Department of Biomedical Laboratory Sciences, Shinshu University School of Medicine, Matsumoto, Japan*
6. *Department of Cellular Transplantation Biology, Kanazawa University Graduate School of Medicine, Kanazawa, Japan*
7. *Department of Hemato-Oncology, International Medical Center, Saitama Medical University, Saitama, Japan*
8. *Division of Public Health, Department of Social Medicine, Saitama Medical University, Saitama, Japan*
9. *Department of Laboratory Medicine, Kawasaki Medical School, Okayama, Japan*
10. *Department of Hematology and Oncology, Kumamoto City Hospital, Kumamoto, Japan*
11. *Department of Hematology, NTT Medical Center Tokyo, Tokyo, Japan*
12. *Department of Medicine and Bioregulatory Science, Graduate School of Medical Sciences, Kyushu University, Fukuoka, Japan*
13. *Department of Hematology, Atomic Bomb Disease and Hibakusha Medicine Unit,*

Atomic Bomb Disease Institute, Nagasaki University, Nagasaki, Japan
14. *Department of Pathology and Tumor Biology, Graduate School of Medicine, Kyoto University, Kyoto, Japan*
15. *Department of Hematology, Nephrology and Rheumatology, Akita University Graduate School of Medicine, Akita, Japan*
16. *Department of Hematology and Oncology, Dokkyo Medical University, Tochigi, Japan*

Naohito Fujishima, MD, naofuji@doc.med.akita-u.ac.jp
Junki Komaru, j-kohmaru@gunma-u.ac.jp
Souichi Koyota, PhD, skoyota@med.akita-u.ac.jp
Keiji Kuba, MD, kuba@med.akita-u.ac.jp
Tomoo Saga, MD, sagatomoo@med.akita-u.ac.jp
Ayumi Omokawa, MD, aomokawa@doc.med.akita-u.ac.jp
Yuki Moritoki, MD, ymoritoki@hos.akita-u.ac.jp
Shigeharu Ueki, MD, shigeh@hos.akita-u.ac.jp
Fumihiro Ishida, MD, fumishi@shinshu-u.ac.jp
Shinji Nakao, MD, snakao8205@staff.kanazawa-u.ac.jp
Akira Matsuda, MD, amatsu@saitama-med.ac.jp
Akiko Ohta, MD, aohta@saitama-med.ac.jp
Kaoru Tohyama, MD, ktohyama@med.kawasaki-m.ac.jp
Hiroshi Yamazaki, yamasaki.hiroshi@cityhosp-kumamoto.jp
Kensuke Usuki, MD, kensuke.usuki@gmail.com
Yasuhiro Nakashima, MD, ynrx3003@intmed3.med.kyushu-u.ac.jp
Shinya Sato, MD, ssato-n@umin.ac.jp
Yasushi Miyazaki, MD, y-miyaza@nagasaki-u.ac.jp
Yasuhito Nannya, MD, ynanya-tky@umin.ac.jp
Seishi Ogawa, MD, sogawa-tky@umin.ac.jp
Kenichi Sawada, MD, kenichi.sawada@mc5.seikyou.ne.jp
Kinuko Mitani, MD, kinukom-tky@umin.ac.jp
Makoto Hirokawa, MD, FACP, makhirokawa08@gmail.com

The first two authors contributed equally to the work.
Correspondence: Naohito Fujishima and Makoto Hirokawa

Address correspondence to: Naohito Fujishima, Division of Blood Transfusion, Akita University Hospital, 44-2 Hiroomote Aza Hasunuma, Akita 010-8543, Japan
Phone: +81-18-884-6313 Fax: +81-18-884-6251
e-mail: naofuji@doc.med.akita-u.ac.jp

Supplementary Figure 1. Sensitivity of the mutation analysis

MDS-L2007 cells were mixed with PBMCs from a healthy control donor at the indicated proportions. Genomic DNAs were extracted from each mixture and then applied to the mutation analysis. The previously known gene mutations harbored by MDS-L2007 cells were detected at 1 or 5%.

				MDS-L2007						
Chromosome	Mutation	Gene_id	Functional consequence	100%	50%	25%	10%	5%	1%	0%
chr20	A -> G	ASXL1	Silent	1.00	0.77	0.54	0.25	0.15	0.06	0.00
chr20	T -> C	ASXL1	Synonymous_S1253S	1.00	0.65	0.36	0.17	0.10	0.02	0.00
chrX	A -> G	ATRX	Silent,Silent	1.00	0.54	0.29	0.12	0.06	0.03	0.00
chrX	T -> C	BCOR	Synonymous_A564A,Synonymous_A564A,Synonymous_A564A,Synonymous_A564A	1.00	0.69	0.45	0.22	0.14	0.03	0.00
chr7	A -> G	CUX1	Synonymous_A422A,Synonymous_A420A	1.00	0.37	0.16	0.06	0.03	0.00	0.00
chrX	G -> A	KDM6A	Synonymous_Q1037Q	1.00	0.53	0.27	0.12	0.07	0.02	0.00
chr17	A -> G	TP53	Silent,Silent,Silent,Silent	1.00	0.48	0.28	0.12	0.06	0.02	0.00
chr17	G -> T	TP53	Silent,Silent,Silent,Silent	1.00	0.54	0.28	0.11	0.08	0.01	0.00
chr17	C -> T	TP53	Silent,Silent,Silent,Silent,Silent,Silent,Silent	1.00	0.57	0.34	0.15	0.08	0.02	0.00
chr11	T -> C	WT1	Synonymous_R157R,Synonymous_R140R,Synonymous_R352R,Synonymous_R369R,Synonymous_R369R	1.00	0.56	0.29	0.11	0.07	0.02	0.00
chrX	T -> G	ZRSR2	Silent	1.00	0.71	0.41	0.23	0.11	0.02	0.00
chrX	T -> G	ZRSR2	Silent	1.00	0.70	0.44	0.22	0.10	0.03	0.00
chrX	T -> C	ZRSR2	Silent	1.00	0.72	0.43	0.22	0.11	0.02	0.00
chrX	C -> T	ZRSR2	Synonymous_N288N	1.00	0.72	0.40	0.18	0.12	0.00	0.00
chr11	C -> CT	CBL	Noncoding	1.00	0.85	0.52	0.25	0.16	0.04	0.00
chrX	AT -> A	ZRSR2	Noncoding	1.00	0.55	0.26	0.13	0.07	0.00	0.00
chr7	G -> A	CUX1	Missense_A464T,Missense_A462T	0.99	0.39	0.18	0.06	0.04	0.01	0.00
chr7	A -> G	CUX1	Silent,Silent	0.99	0.39	0.19	0.06	0.04	0.02	0.00
chr7	G -> A	CUX1	Silent,Silent,Silent	0.99	0.42	0.19	0.08	0.05	0.02	0.00
chr1	C -> G	NRAS	Missense_G12A	0.68	0.43	0.24	0.11	0.06	0.00	0.00
chr19	G -> A	CEBPA	Nonsense_Q311X	0.67	0.44	0.25	0.12	0.07	0.02	0.00
chr7	C -> G	IKZF1	Silent	0.56	0.25	0.15	0.06	0.03	0.00	0.00
chr11	C -> A	HRAS	Silent,Silent,Silent	0.54	0.28	0.14	0.05	0.03	0.00	0.00
chr9	C -> T	CDKN2A	Silent,Synonymous_A167A,Silent,Silent	0.52	0.29	0.16	0.06	0.03	0.00	0.00
chr3	G -> A	MYD88	Synonymous_V206V,Synonymous_V161V,Synonymous_V206V,Silent,Silent	0.51	0.26	0.14	0.06	0.05	0.00	0.00
chr4	C -> G	TET2	Missense_P29R,Missense_P29R	0.50	0.27	0.15	0.05	0.04	0.00	0.00
chr3	G -> T	MYD88	Silent,Silent,Silent,Silent,Silent	0.50	0.29	0.14	0.06	0.04	0.00	0.00
chr4	CT -> C	TET2	Frameshift	0.49	0.27	0.14	0.06	0.04	0.00	0.00
chr9	G -> A	ABL1	Silent,Silent	0.49	0.30	0.18	0.08	0.04	0.00	0.00
chr13	T -> C	FLT3	Synonymous_L561L	0.48	0.28	0.15	0.07	0.04	0.00	0.00
chr8	A -> G	RAD21	Synonymous_A480A	0.48	0.34	0.22	0.10	0.07	0.00	0.00
chr4	A -> C	TET2	Synonymous_P286P,Synonymous_P286P	0.48	0.27	0.14	0.06	0.03	0.00	0.00
chr4	T -> TATAG	TET2	Noncoding,Noncoding	0.42	0.24	0.14	0.06	0.04	0.00	0.00
